# Editorial: Marine microbial-derived molecules and their potential medical and cosmetic applications, volume II

**DOI:** 10.3389/fmicb.2023.1188008

**Published:** 2023-04-04

**Authors:** Jinwei Zhang, Jianxin Wang, Antje Labes, Runying Zeng

**Affiliations:** ^1^State Key Laboratory of Bioorganic and Natural Products Chemistry, Research Center of Chemical Kinomics, Shanghai Institute of Organic Chemistry, Chinese Academy of Sciences, Shanghai, China; ^2^School of Medicine, Xiamen Cardiovascular Hospital Xiamen University, Institute of Cardiovascular Diseases, Xiamen University, Xiamen, Fujian, China; ^3^Hatherly Laboratories, Faculty of Health and Life Sciences, Medical School, Institute of Biomedical and Clinical Sciences, University of Exeter, Exeter, United Kingdom; ^4^Marine Microorganism Ecological and Application Lab, Zhejiang Ocean University, Zhoushan, Zhejiang, China; ^5^Department of Energy and Biotechnology, Flensburg University of Applied Sciences, Flensburg, Germany; ^6^Engineering Innovation Center for the Development and Utilization of Marine Bioresources, Third Institute of Oceanography, Ministry of Natural Resources China, Xiamen, China

**Keywords:** marine microbial ecology, bioactive materials, secondary metabolites, enzymes, oligosaccharides, medical and cosmetic applications

In 2021, a first volume of the Research Topic “*Marine microbial-derived molecules and their potential medical and cosmetic applications*” was published in Frontiers in Microbiology. This volume was comprised of 13 articles covering different aspects of marine microbial diversities, microbial-derived molecules, and their potential medical and cosmetic applications. In this second issue, which groups 8 articles, further insights on these fascinating marine microbial-derived molecules are uncovered.

Marine microbial-derived bioactive compounds and enzymes are attractive for medical and cosmetic applications for several reasons. First, marine microbes inhabit diverse and extreme environments, which has led to the evolution of unique biochemical pathways and biosynthetic capabilities, resulting in the production of novel and diverse bioactive molecules. These molecules can exhibit potent pharmacological and cosmetic properties, such as anti-inflammatory, antioxidant, and antimicrobial activities. Additionally, marine environments remain relatively underexplored compared to terrestrial ecosystems, providing vast opportunities for discovery of new bioactive compounds and enzymes. Finally, the sustainable harvesting of marine microbial-derived compounds and enzymes is possible with minimal environmental impact, making them an attractive alternative to traditional sources of bioactive molecules.

The rise of emerging infectious diseases and multi-drug resistant human pathogens poses a significant global health threat. There is an immediate requirement for new antibiotics to combat bacterial infections that are evolving, especially in the case of gram-negative pathogens. The marine habitat has been established as a promising source for extremophiles, which are potential reservoirs for unique bioactive metabolites. Chen et al. identified two new dibenzopyrones (1, 2) with a rare sulfate group and 10 known compounds (3–12) from the sponge-derived fungal strain *Alternaria* sp. SCSIOS02F49. All compounds exhibited moderate anti-foodborne bacteria activity. Compound 1 was found to alter the external structure of *Staphylococcus aureus* and cause rupture or deformation of the cell membranes, indicating it as the primary antibacterial mechanism. Xu et al. reported a new GH49 dextranase (CeDex) with cold-adaptation and salt-tolerance from a marine bacterium, *Cellulosimicrobium* sp. THN1. Interestingly, CeDex could prevent the formation of *Streptococcus mutans* biofilm and disassemble existing biofilms, therefore having great potential to defeat biofilm-related dental caries. Ribeiro et al. explored the biodiversity of actinobacteria associated with deep-sea sediments collected from the Azores and Madeira regions, and the Arctic Mid-Ocean Ridge, using culture-dependent and independent techniques coupled with metabolomics studies. Crude extracts of 34% of actinomycetes were found to be positive for antimicrobial, anticancer, and anti-inflammatory activities. Further, the annotation of some natural products was made through the analysis of bioactive extracts using dereplication and molecular networking techniques.

Ding et al. reviewed the potential uses and mechanisms of active compounds obtained from marine microorganisms for sun protection, whitening, moisturization, anti-aging, repair, and other applications in cosmetics. The review also addressed the potential challenges and solutions for natural marine-derived products in the cosmetics industry, offering guidance for professionals in the field. Agaro-oligosaccharides have been found to exhibit anti-inflammatory activities. Gu et al. described a culture-independent method to produce a novel β-agarase from the *aga1904* gene, which was obtained from a metagenomic library of Antarctic macroalgae-associated microorganisms. The degradation products of Aga1904 from agarose were mainly agaro-oligosaccharides, showing great effects on key pro-inflammatory markers, including nitric oxide, interleukins 6, and tumor necrosis factor α. Seo et al. identified two new cyclic lipopeptides, cystargamides C and D, from a tidal mudflat-derived isolate, *Streptomyces* sp. JMS132, with antioxidant effects. The entire genome sequence analysis has led to the proposal of the nonribosomal peptide synthetase biosynthetic pathway for the cystargamides, marking the first time such a proposal has been made.

Squalene is widely known for its ability to regulate cholesterol metabolism, prevent tumor growth, and enhance immunity. Zhang et al. evaluated the key factors of cultivation parameters in determining biomass accumulation and squalene production in *Thraustochytrium* sp. ATCC 26185. The squalene yield was 5.37 mg/g cell dry weight by the strain, whereas the production significantly increased to 67.7 mg squalene/g under the optimized conditions.

The tyrosinase enzyme plays a crucial role in the primary immune response in humans, but it can also catalyze undesirable oxidation reactions. Therefore, inhibitors of this enzyme have been extensively sought after. He et al. screened novel tyrosinase inhibitors from marine cyanobacteria and discovered a potent tyrosinase inhibitor called Scytonemin monomer (ScyM), which exhibited a lower IC50 than the commercial inhibitor kojic acid (KA). To evaluate the effectiveness of ScyM, dose-dependent and kinetic studies were conducted, and its effectiveness was compared against related inhibitors such as scytonemin and the methoxy analog ScyM-OMe. Additional synergistic, docking, and cytotoxicity assays also support the potential therapeutic value of ScyM as a tyrosinase inhibitor.

The articles and reviews published in this issue collectively cover a wide range of topics related to biotechnological production of valuable compounds, marine microbial-derived bioactive compounds and enzymes, and their potential medical and cosmetic applications. This issue also highlights the dynamic nature of this field of study and the anticipation of exciting discoveries in the future.

## Author contributions

JZ conceptualized the Research Topic and was responsible for writing the whole passage. JZ, JW, AL, and RZ were responsible for checking and revision. All authors have read and agreed to the published version of the manuscript.

